# Cognitive function and its influencing factors in empty-nest elderly and non-empty-nest elderly adults in China

**DOI:** 10.18632/aging.202416

**Published:** 2021-01-20

**Authors:** Fan Yang, Zhen Li, Guo-Wen Wang, Xiu-Xin Shi, Chang Fu

**Affiliations:** 1 Department of Information Center, Xiangyang No.1 People’s Hospital, Hubei University of Medicine, Xiangyang 441000, Hubei, China; 2 Qinghai Provincial Center for Diseases Prevention and Control, Xining 810010, Qinghai, China; 3 Department of Education, Shandong Provincial Hospital Affiliated to Shandong First Medical University, Jinan 250021, Shandong, China; 4 Office of Medical Quality Control, Qilu Hospital, Cheeloo College of Medicine, Shandong University, Jinan 250012, Shandong, China; 5 Department of Health Psychology, School of Nursing, Cheeloo College of Medicine, Shandong University, Jinan 250012, Shandong, China

**Keywords:** cognitive function, empty-nest elderly, influencing factors, non-empty-nest elderly

## Abstract

Introduction: We investigated cognitive function and its influencing factors in empty-nest and non-empty-nest elderly adults in China.

Results: Cognitive function was better in empty-nest elderly living as a couple but worse in those living alone than in non-empty-nest elderly. Older age, rural habitation, poorer instrumental activities of daily living, and depression were risk factors for cognitive decline, while higher education was protective. Women had poorer cognitive function than men among non-empty-nest elderly and empty-nest elderly living as a couple. Among non-empty-nest elderly, those who were divorced/widowed/never married, underweight or economically active exhibited poorer cognitive function. Having two or more chronic diseases and being overweight were associated with better cognitive function among empty-nest elderly living as a couple.

Conclusion: These findings suggest that cognitive function is poorest in empty-nest elderly living alone and best in empty-nest elderly living as a couple. The factors influencing cognitive function differed according to empty-nest status, which should be considered in interventions.

Methods: 5549 elderly from the 2015 China Health and Retirement Longitudinal Study were included in this study. Cognitive function was evaluated using the Telephone Interview for Cognitive Status, episodic memory tests and visuospatial ability assessments. Factors influencing cognitive function were determined via multiple linear regression analysis.

## INTRODUCTION

At the end of 2019, the number of people aged 60 years and older in China was 254 million, accounting for 18.1% of the total population [1]. China has the largest elderly population in the world, and the World Population Prospects 2019 estimated that the elderly population in China could exceed 400 million people by 2030 [2]. With the social aging process, the number of empty-nest elderly adults is increasing rapidly in China. Empty-nest elderly adults are those without children or those whose children have already left their care [3], and they usually live alone or only with their spouses. Many families in China only have one child because the government implemented a one-child policy in the last 30 years; thus, when the child leaves home (due to work, study, marriage, etc.), his/her aging parents become empty-nest elderly. According to the China Longitudinal Aging Social Survey, empty-nest elderly adults accounted for 47.5% of the elderly population in China in 2016, and are expected to reach 90% by 2030 [4]. Thus, the majority of the elderly will become empty-nest elderly in a few years.

Given their lack of emotional consolation, interpersonal connection and daily care, empty-nest elderly adults tend to suffer from psychological issues such as loneliness, depression, anxiety and pessimism, in what is known as “empty-nest syndrome” [5]. Thus, there may be greater health risks for empty-nest than for non-empty-nest elderly adults. Indeed, previous studies have demonstrated that empty-nest elderly adults have a poorer quality of life [6] and worse mental [7] and physical health [8] than non-empty-nest elderly. The health of this vulnerable population has become an important issue for society and the research community.

Healthy cognitive function is considered an important dimension of successful aging [9]. Declining cognitive function not only seriously impairs older adults’ quality of life, but also increases the burden on families and society [10]. However, there has been little research on cognitive function and the factors influencing it among empty-nest elderly adults in China, and the results of previous studies have been mixed. One study found no significant cognitive differences between empty-nest and non-empty-nest elderly adults in Wuhan, China [11], while another study found that cognitive abilities were impaired in empty-nest elderly adults [8]. In addition, neither of these studies divided empty-nest elderly adults based on whether they lived alone or as a couple. The elderly in these two categories have different living environments, social support structures and care resources, so their cognitive function and the factors influencing it may differ.

In this study, we hypothesized that empty-nest elderly adults would have poorer cognitive function than non-empty-nest elderly adults, and that the factors influencing cognitive function would differ among empty-nest elderly adults living alone, empty-nest elderly adults living as a couple and non-empty-nest elderly adults. We used national data to investigate cognitive function and the factors influencing it in empty-nest and non-empty-nest elderly adults in China.

## RESULTS

### Sample characteristics

Of the 5549 participants (mean age = 68.7 years, standard deviation = 6.8 years), 56.0% were men. In total, 80.0% of the participants lived in rural areas, and 66.2% were married/cohabitating. Among the participants, 52.3% had not received a formal education, and 51.5% reported having a fair health status. More than half of the participants had never smoked or drunk alcohol (51.5% and 64.9%, respectively). A total of 62.0% of the participants had a normal body mass index (BMI), and 55.3% had two or more kinds of chronic diseases. Over 60% of the participants had completely normal basic activities of daily living (BADL) and instrumental activities of daily living (IADL). Of the participants, 91.5% were satisfied with life and 96.0% were satisfied with the relationship with their child. In total, 52.1% of the participants still engaged in economic activities. The depression score was 8.8 ± 6.6 and the overall cognitive score was 9.6 ± 4.3 in the sample. (Table 1).

**Table 1:** Characteristics of the study population and differences between groups.

**Characteristics**	**Total(n=5549)**	**Empty-nest elderly (living alone) (n=1056)**	**Empty-nest elderly (living as a couple) (n=2202)**	**Non-empty-nest elderly (n=2291)**	***χ^2^*/*F***	***P***
Age, mean ± SD	68.7±6.8	70.9±7.3	68.2±6.3	68.2±6.8	70.608	<0.001^a^
Gender (%)					157.974	<0.001^b^
Male	56.0	43.5	65.5	52.7		
female	44.0	56.5	34.5	47.3		
Marital status(%)					2499.824	<0.001^b^
Married/Cohabitating	66.2	9.7	97.6	62.2		
Never married/divorced/ widowed	33.8	90.3	2.4	37.8		
Region (%)					11.938	0.003^b^
Urban	20.0	16.2	20.7	21.1		
Rural	80.0	83.8	79.3	78.9		
Education level(%)					110.598	<0.001^b^
No normal education	52.3	64.5	45.1	53.6		
Elementary school	24.5	20.3	27.4	23.5		
Middle school	14.7	10.1	16.6	15.0		
High school or above	8.5	5.1	10.8	7.8		
Smoking (%)					30.911	<0.001^b^
Current/Past	48.5	43.8	53.0	46.4		
Never	51.5	56.2	47.0	53.6		
Alcohol consumption(%)					36.453	<0.001^b^
Current/Past	35.1	29.6	39.6	33.4		
Never	64.9	70.4	60.4	66.6		
Chronic diseases(%)					1.641	0.801^b^
0	17.4	17.4	16.9	17.8		
1	27.3	27.1	26.9	27.9		
≥2	55.3	55.5	56.2	54.3		
BADL(%)					1.387	0.500^b^
Completely normal	65.4	63.7	65.8	65.9		
Functional decline	34.6	36.3	34.2	34.1		
IADL(%)					57.826	<0.001^b^
Completely normal	63.0	55.2	68.4	61.4		
Functional decline	37.0	44.8	31.6	38.6		
BMI(%)					20.382	0.002^b^
Normal	62.0	65.9	60.2	61.9		
Underweight	7.4	7.1	6.5	8.3		
Overweight	26.2	24.2	27.9	25.5		
Obese	4.4	2.8	5.4	4.3		
Self-rated health (%)					6.048	0.196^b^
Good	20.6	20.2	19.6	21.8		
Fair	51.5	49.9	52.7	51.1		
Poor	27.9	29.9	27.7	27.1		
Life satisfaction (%)					25.618	<0.001^b^
Satisfied	91.5	87.9	93.3	91.5		
Not satisfied	8.5	12.1	6.7	8.5		
Satisfaction with relationship of child (%)					0.409	0.815^b^
Satisfied	96.0	95.7	96.0	96.2		
Somewhat satisfied	4.0	4.3	4.0	3.8		
Economic activity (%)					29.921	<0.001^b^
Yes	52.1	46.2	56.1	51.1		
No	47.9	53.8	43.9	48.9		
Depression, mean ± SD	8.8±6.6	10.4±7.2	8.0±6.3	8.8±6.6	44.683	<0.001^a^
TICS-10, mean ± SD	6.3±2.9	5.5±2.9	6.7±2.7	6.2±3.0	69.804	<0.001^a^
Draw a figure, mean ± SD	0.6±0.5	0.4±0.5	0.6±0.5	0.6±0.5	57.526	<0.001^a^
Words recall, mean ± SD	2.8±1.8	2.4±1.8	3.0±1.8	2.8±1.8	42.030	<0.001^a^
Overall cognitive function, mean ± SD	9.6±4.3	8.3±4.4	10.4±4.0	9.5±4.4	93.601	<0.001^a^

Note: SD, standard deviation; BMI, body mass index; BADL, basic activities of daily living; IADL, instrumental activities of daily living; ^a^*P* values were derived from one-way ANOVA; ^b^*P* values were derived from chi-square test; mean values or distribution were significantly different among the three groups (one-way ANOVA or χ^2^-test; *P* < 0.05).

In our study, 58.7% of the participants were empty-nest elderly. Among them, 32.4% lived alone and 67.6% lived with their spouses or partners. There were significant differences in age, gender, marital status, region, education level, smoking, alcohol consumption, IADL, BMI, life satisfaction, economic activity, depression and cognitive function among the three groups (empty-nest elderly living alone, empty-nest elderly living with a spouse and non-empty-nest elderly), while there were no significant differences in chronic diseases, BADL, self-rated health or satisfaction with the relationship with their child among the three groups. Empty-nest elderly adults living as a couple had the highest cognitive scores among the three groups, while empty-nest elderly adults living alone had the lowest cognitive scores. (Table 1).

### Influencing factors of cognitive function among empty-nest elderly and non-empty nest elderly

In the empty-nest (living alone) group, multiple linear regression analysis indicated that older age (B=-0.119, p<0.001), rural area habitation (B=-1.685, p<0.001), poorer IADL (B=-1.209, p<0.001) and depression (B=-0.073, p<0.001) were associated with a higher risk of cognitive decline. On the other hand, higher education levels (elementary school: B=2.508, p<0.001; middle school: B=3.506, p<0.001; high school and above: B=4.098, p<0.001) were associated with better cognitive function. (Table 2).

**Table 2:** Multiple linear regression analysis on the influencing factors associated with cognitive function among empty-nest elderly and non-empty-nest elderly.

**Characteristics**	**Empty-nest (living alone)**		**Empty-nest (living as a couple)**		**Non-empty-nest**	
	**B(SE)**	***P***	**B(SE)**	***P***	**B(SE)**	***P***
Age	**-0.119(0.017)**	**<0.001**	**-0.140(0.013)**	**<0.001**	**-0.118(0.012)**	**<0.001**
Gender (ref. male)						
Female	-0.368(0.313)	0.239	**-0.705(0.208)**	**0.001**	**-0.877(0.218)**	**<0.001**
Marital status(ref. Married/Cohabitating)						
Never married/ divorced/widowed	-0.170(0.374)	0.649	-0.429(0.464)	0.355	**-0.891(0.167)**	**<0.001**
Region (ref. Urban)						
Rural	-1.685(0.343)	<0.001	-1.282(0.210)	<0.001	-0.976(0.202)	<0.001
Education level(ref. No normal education)						
Elementary school	**2.508(0.317)**	**<0.001**	**2.252(0.183)**	**<0.001**	**2.535(0.187)**	**<0.001**
Middle school	**3.506(0.416)**	**<0.001**	**3.107(0.231)**	**<0.001**	**3.063(0.239)**	**<0.001**
High school or above	**4.098(0.584)**	**<0.001**	**3.420(0.281)**	**<0.001**	**3.781(0.306)**	**<0.001**
Smoking (ref. Current/Past)						
Never	-0.531(0.296)	0.073	0.194(0.183)	0.289	-0.057(0.197)	0.774
Alcohol consumption(ref. Current/Past)						
Never	-0.021(0.256)	0.933	-0.122(0.158)	0.439	0.107(0.168)	0.523
Chronic diseases(ref. 0)						
1	-0.368(0.349)	0.293	0.297(0.226)	0.189	-0.008(0.230)	0.972
≥2	0.326(0.330)	0.324	**0.435(0.214)**	**0.042**	0.287(0.219)	0.189
BADL(ref. Completely normal)						
Functional decline	**-0.138(0.282)**	**0.626**	**0.114(0.195)**	**0.561**	**-0.106(0.180)**	**0.558**
IADL(ref. Completely normal)						
Functional decline	-1.209(0.256)	<0.001	-1.220(0.177)	<0.001	-1.416(0.174)	<0.001
BMI(ref. Normal)						
Underweight	-0.710(0.463)	0.127	-0.013(0.317)	0.968	**-0.666(0.279)**	**0.017**
Overweight	0.290(0.290)	0.319	**0.380(0.167)**	**0.023**	0.206(0.198)	0.302
Obese	0.153(0.650)	0.815	0.470(0.345)	0.175	0.159(0.375)	0.673
Self-rated health(ref. Good)						
Fair	0.42(0.296)	0.888	-0.029(0.193)	0.881	0.365(0.189)	0.054
Poor	0.40(0.360)	0.912	-0.036(0.241)	0.883	0.168(0.243)	0.490
Life satisfaction(ref. Satisfied)						
Not satisfied	-0.063(0.378)	0.868	-0.361(0.298)	0.226	-0.221(0.269)	0.410
Satisfaction with relationship of child (ref. Satisfied)						
Fair	0.094(0.596)	0.875	0.514(0.371)	0.167	0.068(0.386)	0.861
Economic activity (ref. No)						
Yes	0.041(0.255)	0.872	0.088(0.166)	0.596	**-0.368(0.170)**	**0.030**
Depression	**-0.073(0.020)**	**<0.001**	**-0.069(0.014)**	**<0.001**	**-0.054(0.014)**	**<0.001**

Note: B, Coefficient; SE, Standard error; BMI, body mass index; BADL, basic activities of daily living; IADL, instrumental activities of daily living; *P* < 0.05 is statistically significant.

In the empty-nest (living as a couple) group, older age (B=-0.140, p<0.001), female gender (B=-0.705, p=0.001), rural area habitation (B=-1.282, p<0.001), poorer IADL (B=-1.220, p<0.001) and depression (B=-0.069, p<0.001) were associated with a higher risk of cognitive decline. However, completing higher education levels (elementary school: B=2.252, p<0.001; middle school: B=3.107, p<0.001; high school and above: B=3.420, p<0.001), having two or more chronic diseases (B=0.435, p=0.042) and being overweight (B=0.380, p=0.023) were associated with better cognitive function. (Table 2).

In the non-empty-nest group, older age (B=-0.118, p<0.001), female gender (B=-0.877, p=0.001), a marital status of never married/divorced/widowed (B=-0.891, p<0.001), rural area habitation (B=-0.976, p<0.001), poorer IADL (B=-1.416, p<0.001), underweight status (B=-0.666, p=0.017), economic activity participation (B=-0.368, p=0.030) and depression (B=-0.054, p<0.001) were associated with a higher risk of cognitive decline. In contrast, higher education levels (elementary school: B=2.535, p<0.001; middle school: B=3.063, p<0.001; high school and above: B=7.781, p<0.001) were associated with better cognitive function. (Table 2).

## DISCUSSION

With the rapidly aging population, empty-nest will become the main condition of family in China. Therefore, the health of the empty-nest elderly population is a major issue that the government and society cannot ignore. To our knowledge, this is the first study to compare cognitive function and their influencing factors among non-empty-nest elderly adults, empty-nest elderly adults living as a couple and empty-nest elderly adults living alone. We hope that our findings will draw the attention of the government and society to the cognitive health of both empty-nest and non-empty-nest elderly adults so as to improve their quality of life.

Our study revealed that, among the three groups of elderly adults we evaluated, empty-nest elderly adults living alone had the worst cognitive function. This group had an older mean age, a larger proportion of participants living in rural areas, a lower education level, poorer IADL and higher depression scores than the other two groups. Notably, the same characteristics (older age, rural area habitation, a lower education level, poorer IADL and depression) were identified as common risk factors for cognitive functional decline among the three groups, consistent with previous studies [11, 12–14]. Older elderly adults are more likely than younger elderly adults to develop geriatric diseases and to exhibit poor social adaptation, which could reduce their cognitive function [11]. Regarding the location of residence, elderly adults living in rural areas have less access to health care than those living in urban areas. Furthermore, the economic limitations in rural areas reduce access to a variety of entertainment activities. The elderly in rural areas may also be limited to less cognitively demanding occupations, which could lead to poorer cognitive function [14]. A previous study indicated that the lower cognitive function among rural Chinese residents could largely be explained by their lower education levels [15]. Higher education levels may stimulate cognitive abilities such as logical reasoning and abstract thinking, and may also prevent neuronal connection loss or strengthen neuronal associations [16]. In our study, poorer IADL may have been associated with poorer cognitive function because IADL involve more complex functional abilities than BADL, and thus can stimulate cognitive function [17]. The negative association between depression and cognitive function in the three groups could be explained by the evidence that elderly adults with depression exhibit white matter hyperintensity in brain magnetic resonance imaging, which is associated with poor cognition [18, 19].

We also found that empty-nest elderly adults living as a couple had the best cognitive function among the elderly. This result was inconsistent with previous studies [8, 11] and our hypothesis. The reason may be that, with the progress of society and the improvement of living standards for aging adults, the elderly prefer to enjoy their lives. Unlike non-empty-nest elderly adults and empty-nest elderly adults living alone, empty-nest elderly adults living as a couple do not need to undertake the burden of caring for their grandchildren [20] or confront loneliness. Our results demonstrated that empty-nest elderly adults living as a couple had the lowest depression scores, which could have protected their cognitive function.

In our study, although cognitive function differed significantly among the three groups, self-rated health did not. The reason may be that cognitive decline is a slow process that is difficult for the elderly to detect by themselves, and thus may not be captured in a self-rated health assessment representing the subject’s perception of his/her own health. This finding suggests that empty-nest elderly adults living alone should pay greater attention to their cognitive health and undergo cognitive function tests regularly.

Our results indicated that women had a higher risk of cognitive decline than men among empty-nest elderly adults living as a couple and non-empty-nest elderly adults, but not among empty-nest elderly adults living alone. Gender differences in family roles may explain this phenomenon. Previous studies have found that female caregivers tend to perform more personal care tasks than male caregivers [21]. However, female caregivers may feel more distressed than male caregivers, especially if they do not feel very competent at the caregiving task (i.e., identity-relevant stress) [22]. The anxiety and stress of caring for others could reduce cognitive function among elderly women [23, 24]. In contrast, elderly women living alone do not need to care for others, so they may not experience this anxiety and stress.

We also found that being overweight was associated with better cognitive function among empty-nest elderly adults living as a couple, while being underweight was associated with poorer cognitive function among non-empty-nest elderly adults. Similarly, previous studies have found that a higher BMI can reduce depression (and thus enhance cognitive function) in the elderly [25–27]. In traditional Chinese culture, elderly adults with higher BMIs are characterized as having a happy mindset and high self-esteem because being slightly overweight in China is regarded as a sign of wealth [26]. Both a happy mindset and high self-esteem could help the elderly remain healthy [28]. However, we found no significant relationship between BMI and cognitive function in empty-nest elderly adults living alone. A previous study found that the positive association between happiness and cognition was fully mediated by disability and depression [29]. Thus, in our study, the beneficial effects of the “happiness of fat” may have been offset by the poor IADL and depression among empty-nest elderly adults living alone.

In our study, those who had two or more chronic diseases exhibited better cognitive function among empty-nest elderly adults living as a couple, but not among empty-nest elderly adults living alone or non-empty-nest elderly adults. A possible reason is that empty-nest elderly adults living as a couple with more chronic diseases may receive more support and attention from their spouses or living partners than those in the other groups. A previous study found that individuals will reduce their working hours to care for spouses with chronic diseases [30]. The spouse or partner of an older adult is likely or expected to become a more important, core source of social support as other sources of support diminish and the support of a spouse or living partner is an important promoter of health among the elderly [31]. Empty-nest elderly adults living alone are unlikely to receive spousal support, and the spouses of non-empty-nest elderly adults often take care of not only their spouses but also their children or grandchildren [20]. Therefore, both empty-nest elderly adults living alone and non-empty-nest elderly adults receive limited support from spouses or living partners, while empty-nest elderly adults living as a couple are likely to have enough energy and time to focus on their spouses’ or partners’ health. The number of partners providing informal care to meet the daily life needs of chronically ill spouses is rising [32]. The spouses or living partners of elderly adults in poor health often provide meticulous care and promote health-related behaviors such as physical exercise, adequate sleep and healthy diet consumption. These informal forms of care may improve the health and cognitive function of the elderly. Our results also indicated that older adults who had one kind of chronic disease did not have better cognitive function than those who had no chronic diseases among empty-nest elderly adults living as a couple. It may be that elderly adults with multiple chronic conditions increase their spouses’ anxiety more than those with one chronic condition, and thus receive more attention from their spouses [33].

We also found that participation in economic activities did not influence cognitive function in empty-nest elderly adults living alone or as a couple, but was associated with poorer cognitive function in non-empty-nest elderly adults. This was inconsistent with previous reports indicating that participation in economic activities enhanced cognitive function in the elderly [34, 35]. Economic activity is thought to improve the cognitive function of the elderly by expanding their social networks, connections and resources [36]. However, in our study, the average age of the elderly was 68.7 ± 6.8 years, and 80% of the participants lived in rural areas. In China, most of the economic activities of the elderly in rural areas are associated with agriculture, which would not be expected to expand their social networks, connections and resources. Moreover, with the rapid economic and social development in China, work competition is becoming more and more intense. For this reason, many younger adults lack the economic resources and energy to care for their children or themselves, and choose to live with their elderly parents in order to receive financial aid and care from them as “boomerang children” [37]. Therefore, non-empty-nest elderly adults may experience stress from caring for their families, and may passively participate in economic activities, thus negatively impacting their cognitive function [38].

In addition, we found that non-empty-nest elderly adults who were divorced/widowed/never married were at higher risk for cognitive functional decline than those who were married. As mentioned above, the “boomerang child” phenomenon is becoming more common due to high housing prices and low incomes. “A study from Indonesia found that only a few elderly are reliant on next generation for their daily survival and usually the net flow of inter-generational support is downwards from old to young [39]. Another report from China also found that the non-empty-nest elderly are inclined to support their children downwards [40]. In addition to childcare and household tasks, elderly are often the economic pillars of multi-generational families [39]. Although elderly adults may receive some forms of support from children who are living with them, they may not receive more support than they are giving. When non-empty-nest elderly adults lose the support of their spouses, they must care for their adult children or grandchildren by themselves. As a result, non-empty-nest elderly adults may undergo more stress after losing their spouses, which could lead to cognitive impairment.

The limitations of this study are as follows. First, because this was a cross-sectional survey study, the causal relationships between cognitive function and the factors influencing it could not be determined. Second, the survey depended on self-report, so there was a risk of recall bias due to false or inaccurate responses from the participants.

In conclusion, our study demonstrated that, among the three groups of elderly adults we examined, empty-nest elderly adults living alone had the worst cognitive function, while empty-nest elderly adults living as a couple had the best cognitive function. The factors influencing cognitive function differed among the three groups. Older age, rural area habitation, a lower education level, poorer IADL and depression were the common risk factors for poorer cognitive function among the three groups. Women had poorer cognitive function than men among non-empty-nest elderly adults and empty-nest elderly adults living as a couple. Those who had two or more chronic diseases or were overweight had better cognitive function than their counterparts among empty-nest elderly adults living as a couple. Among non-empty-nest elderly adults, those who were divorced/widowed/never married, underweight and economically active were at greater risk for cognitive decline. It is necessary for community doctors to implement targeted interventions to address the factors influencing cognitive function in the elderly according to their living arrangements. In addition, the government and public health departments should pay greater attention to the cognitive health of the elderly (especially empty-nest elderly adults living alone) and improve the health education of this population. Regular cognitive function tests should be performed to prevent cognitive decline in the elderly. Finally, elderly adults should be aware of the health conditions of their spouses or living partners and provide support and informal care to improve their cognitive function.

## MATERIALS AND METHODS

### Study population

The data used for this study were obtained from the 2015 China Health and Retirement Longitudinal Study (CHARLS), a nationally representative survey of Chinese community-dwelling residents. This survey was conducted by the National School of Development (China Center for Economic Research) of Peking University. A multistage sampling method was used, and 21,096 individuals from 12,400 households in 450 village-level units and 150 county-level units participated in the survey. The sample was stratified by urban districts or rural counties within provinces, and by per capita statistics on the gross domestic product. Counties were then sampled and stratified, with probability proportional to the population. Participants aged 60 years and above who could communicate with the investigators were eligible for this study. Elderly adults with neurological diseases and serious psychiatric disorders (such as stroke, Alzheimer’s disease, Parkinson’s disease, brain atrophy) other than depression were excluded from our study. After excluding subjects with no information about age, cognitive function or living status, we enrolled 5549 subjects in our analysis.

We obtained the data by applying to the National School of Development (China Center for Economic Research) of Peking University. All subjects gave their informed consent for inclusion before participating in the study. The original CHARLS was approved by the Ethical Review Committee of Peking University. The secondary analysis of data from CHARLS did not require ethical approval.

### Assessment of cognitive function

In CHARLS, cognitive function was measured using the American Health and Retirement Survey, which included a Telephone Interview for Cognitive Status (TICS-10), figure drawing and word recall [41, 42]. The tests of “TICS-10”, “figure drawing” and “word recall” are performed face-to-face. TICS-10 involved serially subtracting 7 from 100 (up to five times) and providing the date (month, day, year), day of the week and season of the year, with scores ranging from 0 to 10. For the figure drawing, respondents were shown a picture and asked to draw a similar figure. Those who successfully drew the picture received a score of 1, while those who failed to draw the picture received a score of 0. Episodic memory was assessed based on immediate and delayed word recall. For immediate recall, individuals were asked to recall as many words as they could immediately after the interviewers read a list of 10 Chinese nouns. For delayed recall, subjects were asked to recall as many of the original words as possible after four minutes. The episodic memory score was calculated as the average number of immediate and delayed word recalls, and ranged from 0 to 10. The overall cognition score was calculated as the sum of the TICS-10, word recall and figure drawing scores, and ranged from 0 to 21, with higher scores indicating better cognitive function.

### Definition of empty-nest and non-empty-nest

In our study, participants were asked “with whom do you live?”. Those who reported “living with children” were defined as non-empty-nest elderly. Those who reported “living alone or only living with a spouse or partner” were defined as empty-nest elderly. The empty-nest elderly were divided into two groups: empty-nest elderly living alone and empty-nest elderly living as a couple [4].

### Investigation of influencing factors

The demographic characteristics we investigated included age, marital status (married/cohabitating and divorced/separated/widowed/never married), education level (no formal education, elementary school, middle school, high school and above) and community type (urban or rural).

The lifestyle factors we assessed included smoking (current/past, never) and alcohol consumption (current/past, never). In our study, participants were asked by two question about smoking: “Have you ever chewed tobacco, smoke a pipe, smoked self-rolled cigarettes, or smoked cigarettes/cigars” and “Do you still have the habit or have you totally quit”. Participants were also asked by two question about alcohol consumption: “Do you drink any alcoholic beverages in the past year?” and “Do you ever drink any alcoholic beverages in past?”. The “current alcohol consumption” means the elderly drank alcoholic beverages in the past year. The number of chronic diseases was categorized as zero, one, or two or more. Health status was assessed through self-rating on a five-point scale (very poor, poor, fair, good and very good). Self-rated health was categorized as good (including very good and good), fair or poor (including very poor and poor). Depression was assessed using the 10-item Center for Epidemiological Studies Depression scale [43], which has been validated among middle-aged and elderly respondents in China [44]. The scores ranged from 1 to 30, with higher scores indicating higher numbers of depression symptoms [45]. BADL included basic abilities such as eating, dressing, using the toilet, getting in and out of bed, defecating and bathing [46]. IADL included the ability to do daily housework, make a telephone call, cook, take medicine, go shopping and manage finances. There were four possible responses for each task: “can do it by myself”, “have some difficulties”, “need help” and “cannot do it.” [47]. Participants who expressed any difficulty with any item were classified as having BADL/IADL functional decline. Based on the World Health Organization criteria, BMI was classified into one of four categories: underweight (< 18.5 kg/m^2^), normal (18.5-24.9 kg/m^2^), overweight (25-29.9 kg/m^2^) or obese (≥ 30 kg/m^2^) [48].

Other variables we evaluated included participation in economic activities (includes doing agricultural work, doing non-agricultural work, doing employed work, doing self-employed work), life satisfaction (satisfied or not satisfied) and satisfaction with the relationship with a child (satisfied or somewhat satisfied; because only two respondents select “not satisfied,” we combined “not satisfied” with “somewhat satisfied”).

### Statistics analysis

Data were analyzed using the Statistical Package for the Social Sciences version 20.0 (SPSS Inc., Chicago, IL, USA). Characteristics of the overall respondents were described using means and standard deviations for continuous data and percentages for categorical data. Chi-square tests and one-way analysis of variance were used to explore the univariate relationships between age, marital status, community type, education, smoking, drinking, self-rated health, number of chronic diseases, BMI, depression, BADL/IADL, participation in economic activities, life satisfaction, satisfaction with the relationship with a child, and cognitive function in the three groups (empty-nest living alone, empty-nest living as a couple and non-empty-nest). Multiple linear regression analysis was used to examine the factors influencing cognitive function. We carried out multiple imputation using chained equations to address missing data. A p*-*value less than 0.05 was considered statistically significant.
